# Supplementary material for: Four Trials Is Not Enough: The Amount of Prior Audio–Visual Exposure Determines the Strength of Audio–Tactile Crossmodal Correspondence Early in Development
Source: Behav Sci (Basel). 2025 Aug 30;15(9):1184. doi: 10.3390/bs15091184 (PMC12466736; doi:10.3390/bs15091184)
Supplement: Supplementary file 1 [file behavsci-15-01184-s001.zip › behavsci-3575944-supplementary.pdf]

Supplemental Figure S1. Audio-Tactile (AT) Choice Bias for 0, 4, 8, and 16 trials of AT exposure in 6- to 8-years-old participants (re-analyses of data from Experiment 1 of Chow et al., 2021).

Here we analyzed data collected in a related study from our lab (Chow et al., 2021; Experiment 1: 32 AT trials) in a total of 21 6- to 8-year-olds, recruited from the Living Laboratory @ the Museum of Science, Boston, MA. The mean age of children from this earlier study was 7.50 years (range = 6.13–8.77) (see Table 1 from Chow et al., 2021 for detailed demographics).

Data analysis was similar to that of the Experiment 1, in that we considered 4, 8, and 16 trials of prior exposure. For all participants, we designated the first 4, 8, or 16 AT trials as prior exposure, and analyzed the subsequent 16 AT trials to determine the strength of AT associations.

The final dataset included 20 participants (see Chow et al., 2021; Table 1, for Demographics). The result showed that AT association strength was not significantly different from chance, or 0.5, in participants completing 0 trials ($t(19) = .577$, $p = .571$, $g = 2.424$), 4 trials ($t(19) = .617$, $p = .544$, $g = 2.610$), 8 trials ($t(19) = .928$, $p = .365$, $g = 2.761$), 16 trials of AT exposure ($t(19) = 1.926$, $p = .069$, $g = 2.891$).

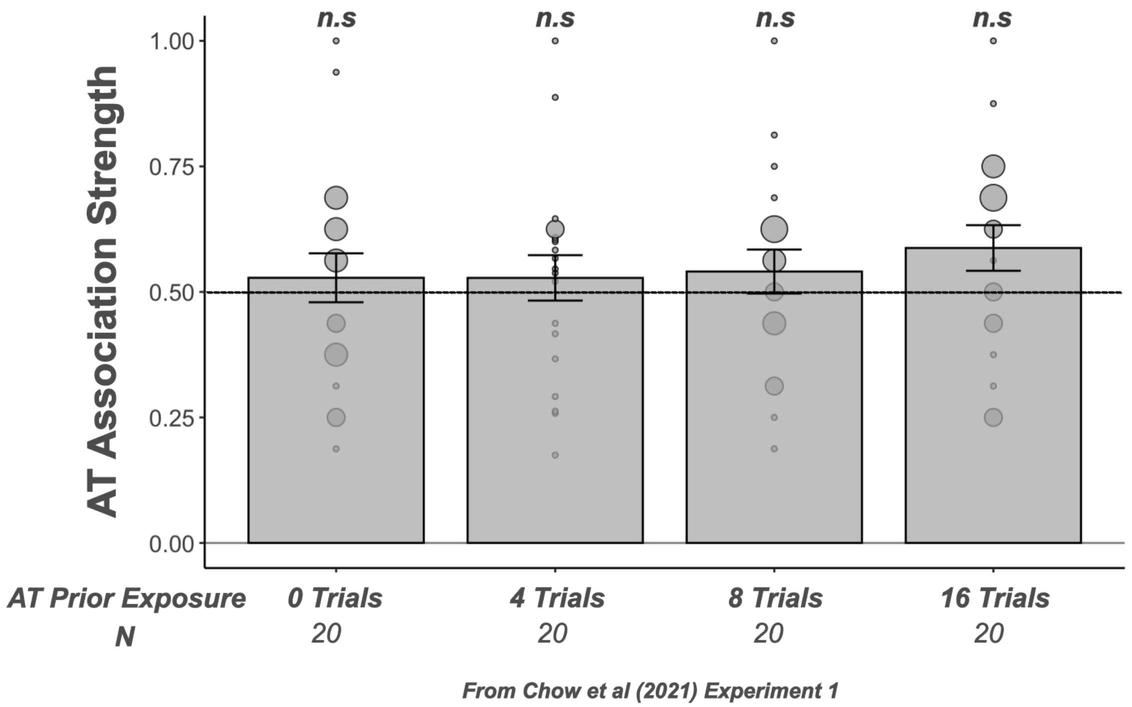

Supplemental Figure S1. Audio-Tactile (AT) Choice Bias for 0, 4, 8, and 16 trials of AT exposure in 6- to 8-years-old participants (re-analyses of data from Experiment 1 of Chow et al., 2021). Individual AT choice bias (dots with size scaled to account for multiple participants at the same value) and mean AT choice bias (bar plot ± standard errors across participants) results show that irrespective of the amount of prior AT exposure, we found no significant effect on subsequent AT choice bias (0 trials: ($t(19) = .577$, $p = .571$, $g = 2.424$); 4 trials: $t(19) = .617$, $p = .544$, $g = 2.610$; 8 trials: $t(19) = .928$, $p = .365$, $g = 2.761$; 16 trials: $t(19) = 1.926$, $p = .069$, $g = 2.891$).

Supplemental Table S1. Fixed effects of the null mixed-effect logistic regression model predicting binary response using number of AV exposure as predictors (95% Wald confidence interval).

Model 1: Binary Choice ~ Intercept

**Fixed effects**	**Coefficient**	**Odd Ratio**	**SE**	***z* value**	***p* value**	**95% confidence interval**	
						**Lower**	**Upper**
**(Intercept)**	.625	1.868	.134	4.665	<.001	.364	0.903

Supplemental Table S2. Comparison of all mixed-effect logistic regression models fit predicting binary response using number of AV exposure trials as predictors

Model	N of parameter	AIC	BIC	logLike	Deviance	Chisq	Df	*p* value
**Binary Choice ~ Intercept**	2	1233.7	1243.5	-614.86	1229.7			
**Binary Choice ~ Intercept + AV exposure group**	3	1230.0	1244.6	-611.99	1224.0	5.724	1	0.017

Supplemental Table S3. Fixed effects of the final mixed-effect logistic regression model predicting binary response using sound category and number of AT exposure trials as predictors (95% Wald confidence interval).

We performed mixed-effect logistic regression modeling to predict participants' odds of choosing a round shape when a given sound was presented and based on the amount of prior AT exposure (4 trials or 8 trials). The alternative model (Akaike information criterion [AIC] = 867.9, Bayesian information criterion [BIC] = 881.4, log likelihood = -431.0) did not improve model fit, $x^2(1) = .013$, $p = .910$, compared with the null model (AIC = 865.9, BIC = 874.9, log likelihood = -431.0).

**Fixed effects**	**Coefficient**	**Odd Ratio**	**SE**	***z* value**	***p* value**	**95% confidence interval**	
						**Lower**	**Upper**
**(Intercept)**	0.214	1.239	0.210	1.018	0.309	-0.211	0.654
**AT exposure group**							
**1: 8 Trials**<br>**0: 4 Trials**	0.019	1.019	0.166	0.113	0.910	-0.308	0.345

Supplemental Table S4. Replicate Analysis in Graven and Desebrock (2018) for Experiment 1 Data, Chow et al., 2021).

Of note, Graven and Desebrock (2018) using a different analysis and number of trials had found that blind adults initially showed no AT associations on Trial 1, but AT associations emerged with repeated AT exposure of the same shape pair, with stronger performance on later trials, 5 and 8, compared to earlier trials, 1 and 4. Interestingly, unlike blind adults, blindfolded, sighted, adults showed worse performance on later trials (Graven & Desebrock, 2018). To ensure that we did not miss changes in AT performance since our paradigm considered a different number of trials and a different analysis, comparing up to 16 AT exposure trials with 16 AT test trials, we replicated the accuracy analysis used in Graven and Desbrock for our data in typically sighted 6- to 8-year-olds.

	**Trial 1**	**Trial 4**	**Trial 5**	**Trial 8**	**Chance Level**
**Correct**	11	12	12	11	10.5
**Incorrect**	10	9	9	10	10.5

Supplemental Table S4 shows the number of participants correctly matching one of the two touched shapes to the corresponding nonsense sound in Trials 1, 4, 5, and 8. A chi-square goodness-of-fit test showed no instant AT association on trial 1, no effects significantly above chance ($x^2(1) = .048$, $p = .827$). Furthermore, there was no evidence of strengthening AT associations, no effects significantly above chance, on trial 4 ($x^2(1) = .429$, $p = .513$), trial 5 ($x^2(1) = .429$, $p = .513$), or trial 8 ($x^2(1) = .048$, $p = .827$). Thus, the mechanisms causing young children to fail to learn from repeated AT exposure differ from those allowing blind adults to improve with repeated AT exposure or allowing blindfolded sighted adults to worsen with repeated AT exposure.

References

Barnard, G. A. (1945). A New Test for 2 × 2 Tables. *Nature*, *156*(3954), 177–177. https://doi.org/10.1038/156177a0

Barnard, G. A. (1947). Significance tests for 2 X 2 tables. *Biometrika*, *34*(1–2), 123–138. https://doi.org/10.1093/biomet/34.1-2.123

Erguler, K. (2016). *Barnard: Barnard's Unconditional Test* (Version 1.8) [Computer software]. https://cran.r-project.org/web/packages/Barnard/index.html
